# An Energy-Efficient FPGA-Based Real-Time IMDD OFDM-PON Enabled by an Efficient FFT

**DOI:** 10.3390/s25237302

**Published:** 2025-12-01

**Authors:** Zhe Zheng, Tianyang Chen, Yuanzhe Qu, Zhengjun Xu, Yingying Chi, Xin Wang, Junjie Zhang

**Affiliations:** 1 Beijing Smartchip Microelectronics Technology Company Limited, Beijing 100192, China; zhengzhe@sgchip.sgcc.com.cn (Z.Z.); chiyingying@sgchip.sgcc.com.cn (Y.C.); 2 Key Laboratory of Specialty Fiber Optics and Optical Access Networks, Joint International Research, Laboratory of Specialty Fiber Optics and Advanced Communication, Shanghai University, Shanghai 200444, China; tyral_chen@shu.edu.cn (T.C.); quyuanzhe@shu.edu.cn (Y.Q.); xuzj@shu.edu.cn (Z.X.); 3 Tianjin Center of National Digital Switching System Engineering and Technological R&D Center, Information Technology Innovation Center of Tianjin Binhai New Area, Tianjin 300457, China

**Keywords:** FFT, IMDD OFDM-PON, energy-efficient, real-time DSP, FPGA

## Abstract

For the first time, a highly energy-efficient 32-parallel 64-point FFT scheme for IMDD OFDM-PON is proposed and implemented on a Xilinx ML605 platform. By experimentally verifying the power consumption model for the FPGA logic resources utilized in the FFT, the relationship between FFT calculating consumption and FPGA logic resource usage is established. Based on this relationship, we derive a resource selection principle for the FFT bit resolution optimization to minimize power consumption under different levels of received optical power. Consequently, the proposed FFT achieves a 76.1% reduction in power consumption compared to the traditional Spiral FFT at a received optical power of −21 dBm. Based on the proposed FFT, the real-time OFDM-PON receiver power consumption can save up to 43% compared with traditional OFDM-PON system.

## 1. Introduction

From 2010 to 2015, the power dissipation of the Information and Communication Technology (ICT) sector was approximately 20 GW and has risen to 1.5 TW by 2025, which would further account for 14% of global greenhouse gas emissions by 2040 [1,2]. Moreover, access networks are responsible for 70% of the energy consumed within the ICT sector [3]. Consequently, developing energy-efficient access network technologies has become a critical research direction. Owing to its advantages, including low power consumption and high reliability, Passive Optical Network (PON) technology has emerged as a leading solution for access networks [4].

Owing to its high spectral efficiency, flexible bandwidth allocation, and robust dispersion tolerance, Orthogonal Frequency Division Multiplexing Passive Optical Network (OFDM-PON) has attracted significant attention for Next-Generation Passive Optical Network (NG-PON) systems [5,6,7,8,9,10]. Among various OFDM-PON schemes, Intensity Modulation–Direct Detection (IMDD) OFDM-PON has become a research focus due to its simplicity and ease of implementation. However, in comparison to traditional Time Division Multiplexing Passive Optical Network (TDM-PON), IMDD OFDM-PON requires more complex analog-to-digital converters/digital-to-analog converters (ADCs/DACs) and digital signal processing (DSP), resulting in higher overall energy consumption for the system [6]. Therefore, research on realizing energy-efficient IMDD OFDM-PON systems is imperative.

A significant challenge arises from the frequency disparity between the FPGA, which typically operates at a few hundred MHz, and the ADC in an IMDD OFDM-PON system, which requires a sampling rate of several GHz. This disparity necessitates high-throughput FFT implementations that consume substantial hardware resources [10]. The FFT can occupy over 80% of the total FPGA logic resources and account for more than half of the system’s dynamic power consumption [8]. Thus, reducing FFT power consumption is pivotal for realizing energy-efficient IMDD OFDM-PON systems.

A direct approach is the minimization of FFT logic resource utilization, and several studies have explored this along several paths. Bouziane et al. [11] prove that FFT power consumption increases with its size. Kimura et al. [12] show that for a fixed FFT size, reducing the computational precision effectively lowers the power consumption. They propose an energy-efficient scheme based on dynamic signal-to-noise ratio (SNR) management to adaptively control the FFT calculation accuracy based on the transmission distance of each optical network unit (ONU). Subsequently, in [13], Kimura et al. combine dynamic SNR management with adaptive modulation and conducted an experimental demonstration. The results show that this scheme could improve energy efficiency for short-distance ONUs by 58.7%. Besides, Hu et al. [6] propose a time-domain interleaved OFDM technique, achieving energy savings of 17% and 26.7% by configuring the sampling-rate-to-FFT-size ratio to 1/2 and 1/4, respectively. An FFT stage-dependent clipping bit-resolution optimization strategy is proposed and experimentally verified by analyzing the dynamic range of FFT calculations, effectively saving 30% of FPGA logic resources [14]. Further, a mathematical model is developed for bit-resolution optimization using probability analysis and provided a simplified method for generating an FFT bit-resolution mapping table [15]. To further minimize the FPGA chip power consumption, a clock-gating technique is proposed to control the clock of the OFDM demodulation module according to the recognized ONU LLID [9]. Previous research has predominantly focused on reducing the FFT size and quantization bit width for full-parallel FFT. However, the existing literature lacks detailed analysis of the power consumption of each module through a high-fidelity power consumption model.

To address this gap, in this paper, we investigate a real-time IMDD OFDM-PON receiver implemented on a Xilinx ML605 platform, employing a 32-parallel 64-point Cooley-Tukey Radix-2 Decimation-In-Time (DIT) FFT (hereafter referred to simply as FFT). We develop and experimentally validate a high-fidelity power consumption model for this FFT, with power estimation based on the minimum unit of FPGA logic resource. Using this model, we establish the relationship between FFT power consumption and FPGA logic resource utilization. This relationship, in turn, enables the derivation of an FFT resource selection principle aimed at minimizing power consumption. By applying this principle—dynamically configuring the FFT bit resolution according to the received optical power and leveraging the system’s inherent characteristics—our proposed FFT achieves a 76.1% reduction in power consumption compared to the traditional Spiral FFT [16] core at a received optical power of −21 dBm. Under this condition, the FFT no longer constitutes the primary source of power consumption within the DSP section of the FPGA-based real-time IMDD OFDM-PON system.

## 2. Operation Principle

### 2.1. FPGA Power Consumption Model for OFDM-PON

As delineated in Equation (1), the total power consumption P of an FPGA chip comprises static $P_{static}$ and dynamic components $P_{dynamic}$ [17]. Since static power consumption is determined by the FPGA manufacturing process, supply voltage, and ambient temperature, this work focuses on analyzing the dynamic power consumption attributable to the DSP algorithms implemented for the OFDM-PON system. The dynamic power consumption is governed by signal toggle rate α within a clock cycle, load capacitance $C_{L}$, supply voltage $V_{dd}$ and operating clock frequency $f_{clk}$. Unless otherwise specified, the term “power consumption” in this paper refers to the dynamic power consumption of the FFT, as it is the pivotal DSP algorithm in the real-time OFDM-PON system.

(1) $${P=P}_{static}+P_{dynamic}=P_{static}+\alpha C_{L}V_{dd}^{2}f_{clk}\;$$

Different with the FFT module by using online Spiral DFT/FFT IP Core Generator provided by Carnegie Mellon University [9], this study exclusively employs a 32-parallel 64-point Cooley-Tukey radix-2 DIT FFT, which is implemented on the FPGA using a pipelined architecture. Leveraging the logic resources of the Xilinx Virtex-6 FPGA on the ML605 board [8], we develop the precise power consumption model for the IMDD OFDM-PON real-time receiver, as presented in Equation (2).

(2) $$P_{FFT}=\frac{\left(\alpha N_{FF}+\beta N_{LUT}+\eta N_{LUTRAM}{+\theta N}_{BRAM}+\delta N_{DSP}\right)f_{clk}}{125}\;$$

where $N_{FF}$, $N_{LUT}$, $N_{LUTRAM}$, $N_{BRAM}$, $N_{DSP}$ represent the numbers of FF, LUT, LUTRAM, block memory and DSP Slices, respectively, for the Xilinx Virtex-6 FPGA. And α, β, η, θ, δ represents the associate coefficients for the dynamic power consumption model.

Through Xilinx Power Estimator (XPE) software simulations [18] and extensive experimental measurements, the fitting coefficients for the models are determined, as summarized in Table 1. These coefficients exhibit close agreement with experimental data. It reveals that the block memory (BRAM) consumes significantly more power than other FPGA logic resources. Therefore, to minimize power consumption in high-throughput FFT implementations on FPGA-based platforms, the use of block memory should be minimized.

### 2.2. Block Memory-Efficient Architecture for Half-Parallel FFT

**Figure 1:**
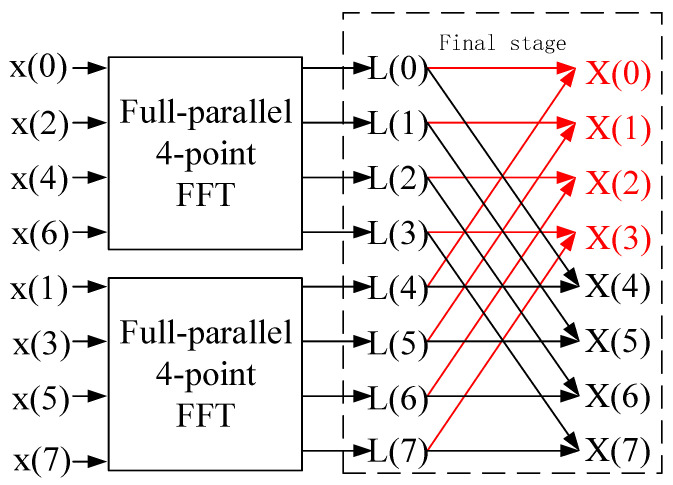
The butterfly operation for 8-point FFT using 4-point FFT.

As demonstrated in our previous work [14], the implementation of a full-parallel *N*-point FFT utilizes only LUT resources and avoids the use of block memory. Consequently, an FPGA-based half-parallel FFT with N/2-parallel *N*-point FFT where *N* is set to 64 in this paper can consume less block memory if a full-parallel N/2-point FFT is used for the FPGA realization of the half-parallel FFT. It is well known that the *N*-point FFT is defined as

(3) $$X\left(k\right)=\sum_{n=0}^{N-1}x\left(n\right)\cdot W_{N}^{nk},k=0,\;1\ldots ,\;N-1\;$$

where $x\left(n\right)$ and $X\left(k\right)$ denote the time-domain and frequency-domain sequences, respectively, and $W_{N}^{nk}$ is the twiddle factor. Derived from Equation (1), Figure 1 illustrates the implementation of a full-parallel 8-point FFT based on the Radix-2 architecture. The full-parallel 8-point FFT structure is constructed by decomposing it into two 4-point FFTs. This is achieved by reordering the input sequence and performing a final butterfly operation at the final stage.

**Figure 2:**
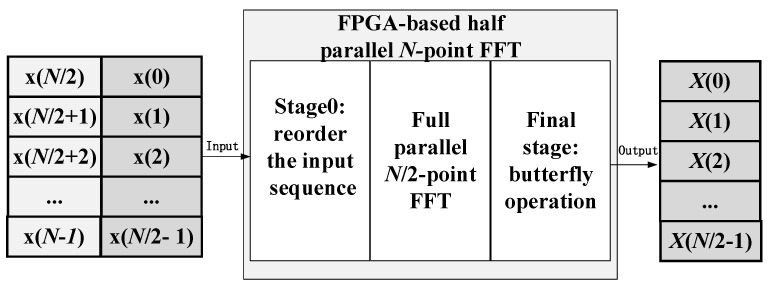
The realization scheme of the half parallel N-point FFT using the full parallel N/2-point FFT.

Therefore, the implementation scheme for the half-parallel *N*-point FFT, with an input parallelism of N/2, is depicted in Figure 2. The proposed FPGA-based half-parallel *N* -point FFT architecture comprises three primary processing modules. The first module (Stage 0) reorders the input sequence into a bit-reversed order to facilitate the correct natural-order output of the final *N*-point FFT. The second module performs the full-parallel N/2-point FFT calculation, which, as established in our prior work [14], operates without using block memory. The third and final module carries out the butterfly calculations, highlighted by the red lines in Figure 1, to generate the *N* output points. Critically, as evidenced by the dataflow in Figure 1, the reordering processing essential for the half-parallel *N*-point FFT is implemented using only FPGA slice register (SR) resources, entirely avoiding the use of block memory. The detailed FPGA implementation is discussed in Section 3.

## 3. Energy-Efficient Scheme for the Half-Parallel 64-Point FFT

### 3.1. Overall Architecture of the FPGA-Based Real-Time IMDD OFDM-PON Energy-Efficient Half Parallel 64-Point FFT Design

A half-parallel 64-point FFT architecture is employed to meet the 32-parallel processing requirement of the real-time OFDM-PON experimental platform described in Section 4. This design offers an optimal balance between implementation complexity and performance. The detailed design of the FFT is elaborated in the following subsections.

#### 3.1.1. Stage 0 Processing Module: Input Reordering

**Figure 3:**
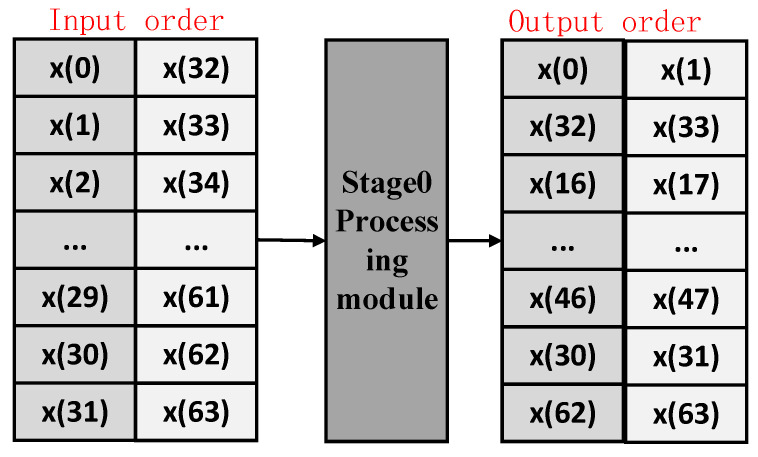
The output order description for the stage0 processing module.

As outlined in Section 2.2, the function of the Stage 0 processing module is to reorder the input sequence. The specific output order for a half-parallel 64-point FFT is illustrated in Figure 3 and is defined by the following sequences.

The detailed output order for the first 32 outputs shall be arranged in accordance with the input sequence as follows: [0, 32, 16, 48, 8, 40, 24, 56, 4, 36, 20, 52, 12, 44, 28, 60, 2, 34, 18, 50, 10, 42, 26, 58, 6, 38, 22, 54, 14, 46, 30, 62]. And the detailed output order for the last 32 outputs shall be arranged in accordance with the input sequence as follows: [1, 33, 17, 49, 9, 41, 25, 57, 5, 37, 21, 53, 13, 45, 29, 61, 3, 35, 19, 51, 11, 43, 27, 59, 7, 39, 23, 55, 15, 47, 31, 63].

**Figure 4:**
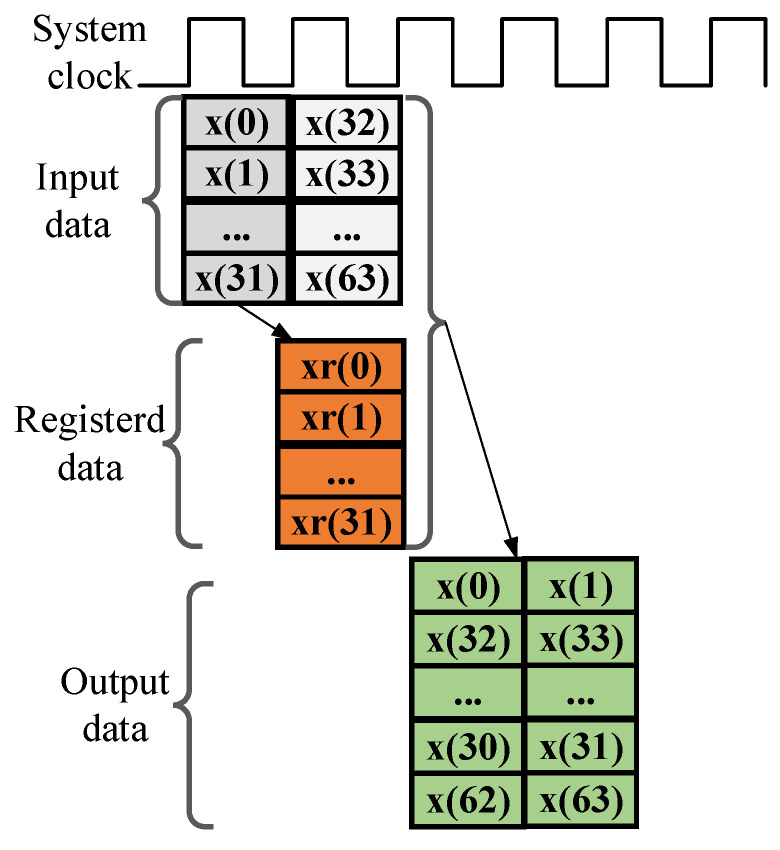
The FPGA-based realization timing sequence for the stage0 processing module.

The FPGA implementation for generating the required output order is depicted in the schematic of Figure 4. The first 32 samples $x\left(0\right)\sim x(31)$ for the input of the 32-parallel 64-point FFT are initially stored in an internal register array $xr\left(0\right)\sim xr(31)$. These temporarily stored samples are then interleaved with the subsequent 32 input samples to produce the correctly ordered sequence for the next stage of FFT computation.

#### 3.1.2. Final Processing Module: Output Combination

**Figure 5:**
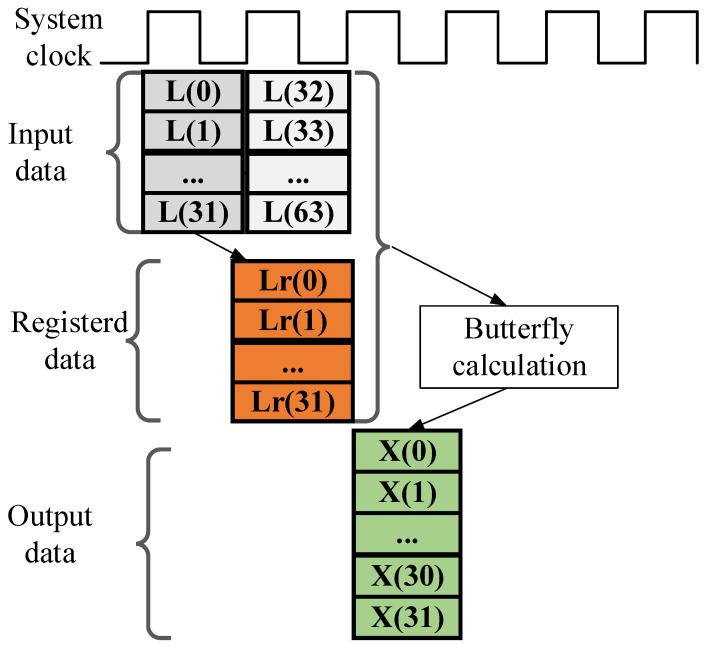
The FPGA-based realization timing sequence for the final stage processing module.

A full-parallel 32-point FFT core is utilized to construct the half-parallel 64-point FFT, which has been used in paper [14]. Thus, to achieve the final calculation of the half-parallel 64-point FFT, the corresponding FPGA implementation for the final output combination is detailed in Figure 5. The computation of the final 64-point FFT outputs proceeds as follows: the first 32 output data $L\left(0\right)\sim L(31)$ of the output for the full parallel 32-point FFT is firstly registered into the internal data $Lr\left(0\right)\sim Lr(31)$, and the generated $Lr\left(0\right)\sim Lr(31)$ data can be combined with the last 32 output data $L\left(32\right)\sim L(63)$ to generate the 64-point FFT result according the following butterfly calculation equation:

(4) $$X\left(n\right)=L\left(n+32\right)+Lr\left(0\right)*W_{64}^{n},\;n=0,\;1,\;2\ldots 31$$

A key advantage of this design, as evidenced by the data path in Figure 5 and the operations in Equation (4), is that the final processing module does not require any block memory.

### 3.2. System-Level Energy-Efficient Solution for FPGA-Based Real-Time IMDD OFDM-PON Receiver

**Figure 6:**
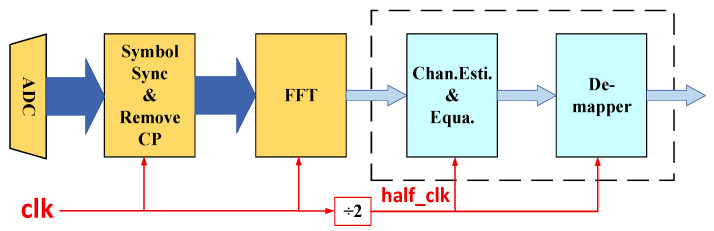
The system-level energy saving scheme using two clocks.

The inherent Hermitian symmetry in IMDD OFDM-PON systems implies that the effective data throughput is reduced by half following the FFT operation at the receiver. This characteristic creates an opportunity to lower the processing clock frequency by half for the subsequent demodulation stages, achieving significant energy savings without compromising the system performance. The proposed system-level energy-efficient solution, which implements this dual-clock strategy, is depicted in Figure 6. A new clock domain, half_clk, operating at the half the frequency of the primary system clock clk, is introduced. This lower-frequency clock drives the demodulation stages subsequent to the FFT, the details of which are presented in Section 4. Consistent with the traditional FPGA-based OFDM receiver demodulation procedure, the Symbol Sync, Remove CP and FFT modules remain in the high-frequency clk clock domain to ensure real-time processing. Consequently, the proposed dual-clock method significantly reduces the overall power consumption of the FPGA-based real-time IMDD OFDM-PON receiver compared to a traditional single-clock design, while maintaining identical system performance.

## 4. Experimental Setup

**Figure 7:**
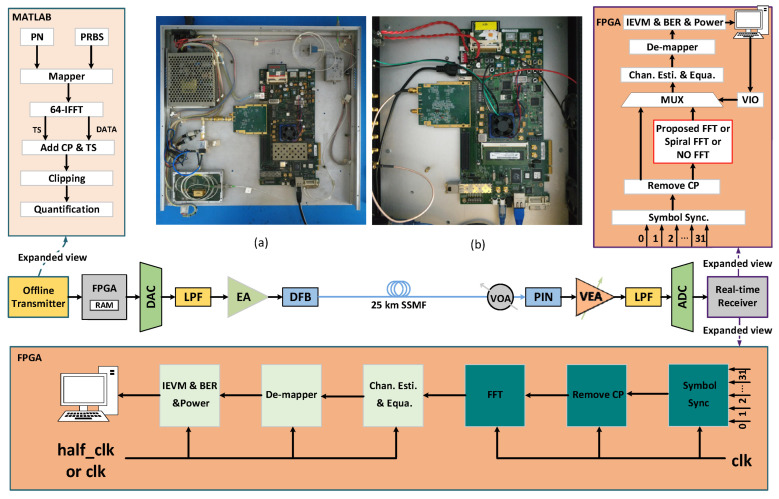
FPGA-based real-time IMDD OFDM-PON receiver. (**a**) Transmitter based on Xilinx ML605 FPGA board; (**b**) real-time receiver based on Xilinx ML605 FPGA board.

To validate the proposed energy-efficient FFT scheme and system-level solution, a real-time IMDD OFDM-PON platform is implemented using two Xilinx ML605 FPGA boards, as shown in Figure 7. The key system parameters are summarized in Table 2 which are also used in the paper [9].

At the transmitter side, the OFDM modulation is performed offline using MATLAB R2021a software. This process includes pseudo-random binary sequence (PRBS) generation, constellation mapping, IFFT operation, insertion of a cyclic prefix (CP) and training sequence (TS), as well as quantization and clipping. The FFT/IFFT size is 64, and the modulation format is configurable among BPSK, 4-QAM, 8-QAM, 16-QAM, 32-QAM, and 64-QAM. To mitigate the channel’s low-pass frequency response and ADC roll-off effects, data is transmitted on 30 subcarriers (indices 1 to 31). To comply with the requirements of an IMDD system, subcarriers 32 to 63 are configured to enforce Hermitian symmetry. Following the 64-point IFFT, a complete OFDM frame is assembled by inserting a 16-sample cyclic prefix and incorporating specific synchronization and control fields, including 80 zeros, two ‘1’ bits, one LLID, two TS symbols, and 100 data-carrying OFDM symbols, as illustrated in Figure 7. The assembled frame is then subjected to 12-bit quantity and clipped at approximately 12 dB. The resulting digital signal is transferred via the UDP protocol to the RAM of the ML605 FPGA board, which is equipped with a 4 GS/s, 12-bit DAC, as shown in Figure 7a. The resulting analog electrical signal from the DAC drives a narrow-bandwidth distributed feedback laser (DFB-LD) through a chain comprising a 2 Vpp variable attenuator and a 13 dB amplifier. This modulates the baseband OFDM signal onto an optical carrier, which is subsequently transmitted over 25 km of standard single-mode fiber (SSMF).

At the receiver side, a variable optical attenuator (VOA) first adjusts the received optical power. The signal is then converted from the optical to the electrical domain using a 2.7 GHz PIN photodetector. Subsequently, a variable-gain electrical amplifier (VGA) amplifies the OFDM signal to fully utilize the dynamic range of a 10-bit analog-to-digital converter (ADC), which digitizes the received analog signal. The digitized signal is demultiplexed into 32 parallel streams for processing. Two distinct FPGA implementations are employed to validate the proposed schemes.

Implementation A: This design employs a synchronization header (Syn header) for timing recovery. Subsequent processing involves frame synchronization, removal of the cyclic prefix (CP) and training sequences (TSs), followed by FFT operation (using either the traditional Spiral FFT, the proposed FFT, or bypassed for reference). Channel estimation, channel equalization, and symbol demapping are then performed to demodulate the OFDM symbols. Finally, the recovered bits are compared with the original PRBS in a real-time bit error rate (BER) tester to evaluate performance.

Implementation B: This design implements the proposed dual-clock strategy. The Symbol Sync, Remove CP, and FFT modules operate in the high-frequency clk domain to maintain real-time throughput. The subsequent DSP stages—Channel Estimation, Equalization, Demapping, and the Inverse Error Vector Magnitude (IEVM), BER, and Power monitoring modules—are placed in the half_clk domain, which operates at half the frequency of clk. All other components are identical to Implementation A.

To accurately measure the FPGA’s power consumption, the digital power rails are monitored in real-time via the UCD9240 power controller on the ML605 board. This is achieved using a USB-TO-GPIO adapter that reads power data through the PMBus command protocol. To isolate the power consumption attributable solely to the FFT module, a Virtual Input/Output (VIO) core is employed. The VIO holds the input to the stages subsequent to the FFT constant, thereby preventing any variation in their power consumption from affecting the measurement of the FFT block itself. The power consumption of the FFT block is then determined by comparing the total system power dissipation under three conditions: when employing the traditional Spiral FFT, when employing the proposed FFT, and when the FFT is bypassed (No FFT).

## 5. Experimental Results

### 5.1. Real-Time Experimental Verification of the BER Performance of the Proposed FFT

#### 5.1.1. Selection of BER Performance Indicator and FFT Bit Resolution

As verified in the references [8,9], the 16-bit quantified Spiral FFT on this platform achieves performance virtually identical to a floating-point reference. Therefore, it is adopted herein as the baseline for BER performance comparison. According to the impact of quantization precision in a 64-point FFT on IMDD OFDM-PON system performance across varying received optical power (ROP) levels [14,15], it is concluded that quantizing the twiddle factors to 9 bits degrades the overall system’s Inverse Error Vector Magnitude (IEVM) performance by merely 0.1 dB. Based on this finding, a twiddle factor word length of 9 bits is employed in this work. Furthermore, the minimum required bit width for each FFT stage at different ROP levels is summarized in Table 3. The proposed FFT architecture uses this table as the guideline for dynamic bit-resolution selection.

#### 5.1.2. Comparative BER Performance of Spiral FFT and Proposed FFT

**Figure 8:**
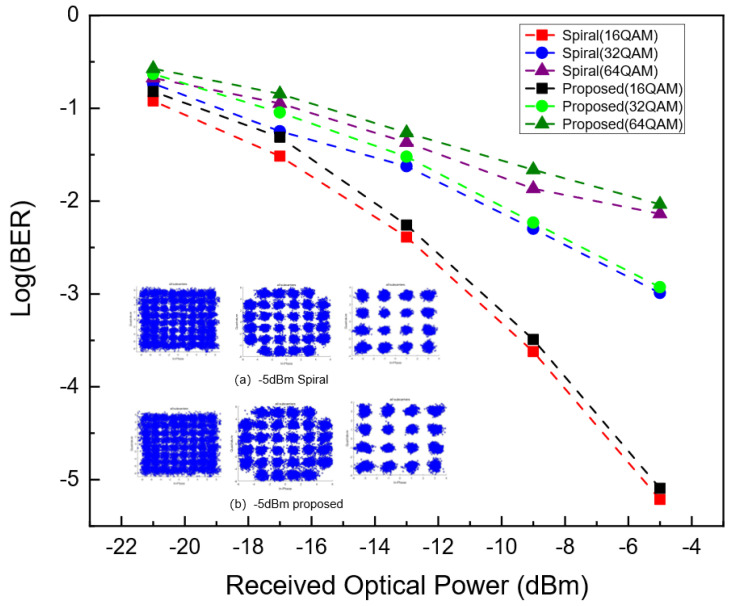
The BER performance against the optical received optical power. (**a**) The constellation diagrams for Spiral; (**b**) The constellation diagrams for this paper.

To validate that the proposed FFT achieves bit error rate (BER) performance comparable to that of the Spiral FFT across various modulation formats and received optical power (ROP) levels, we measure the BER performance of both the Spiral FFT and the proposed FFT. The results were obtained using 16-QAM, 32-QAM, and 64-QAM modulation formats over a range of ROP values typical for PON systems: −5, −9, −13, −17, and −21 dBm. As shown in Figure 8, the results demonstrate that the BER performance of the proposed FFT exhibits a degradation of less than 1% compared to the Spiral FFT across all tested conditions, confirming that the two implementations deliver nearly identical performance. To further corroborate this finding, constellation diagrams for all subcarriers are captured at a ROP of −5 dBm for both the Spiral FFT and the proposed FFT under different modulation formats, as illustrated in insects (a) and (b) of Figure 8. A visual comparison reveals that the constellation points of the proposed FFT exhibit a nearly identical degree of dispersion to those of the Spiral FFT, indicating comparable signal fidelity.

#### 5.1.3. BER Performance of the Proposed FFT with Adaptive Modulation

**Figure 9:**
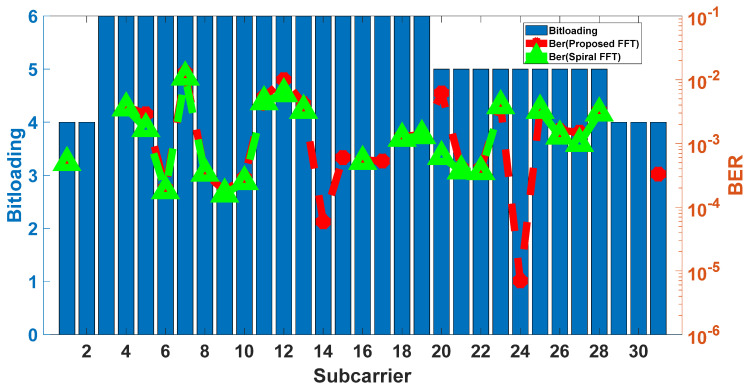
Adaptive modulation format and BER performance for Spiral FFT and proposed FFT.

To assess the system performance under realistic operating conditions, in which adaptive modulation format is always employed to compensate for varying received optical power and system frequency roll-off, the BER of both the Spiral FFT and the proposed FFT is evaluated using an adaptive modulation scheme at a received optical power of −5 dBm. As shown in Figure 9, the proposed FFT and the Spiral FFT achieve nearly identical BER performance under adaptive modulation.

### 5.2. Real-Time Experimental Verification of the Energy-Efficient Effect of Proposed FFT

**Figure 10:**
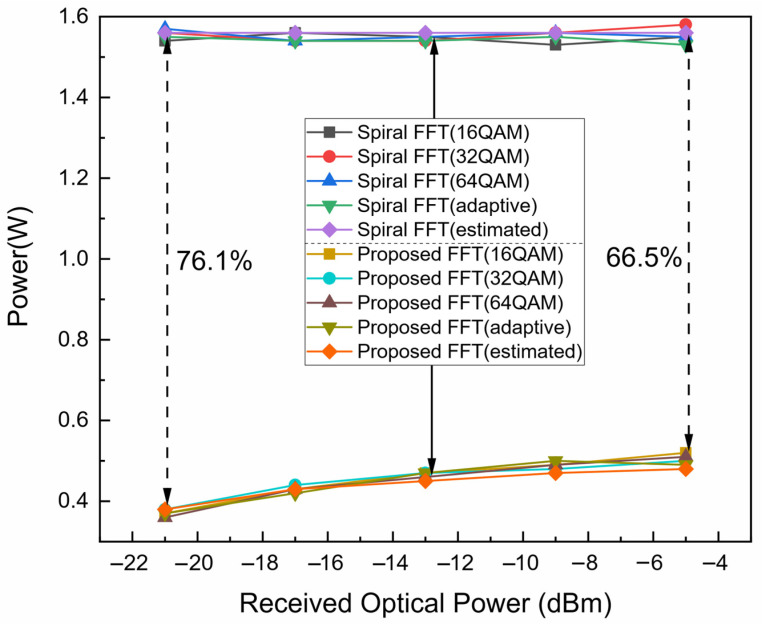
Power consumption versus received optical power for various FFT schemes.

**Figure 11:**
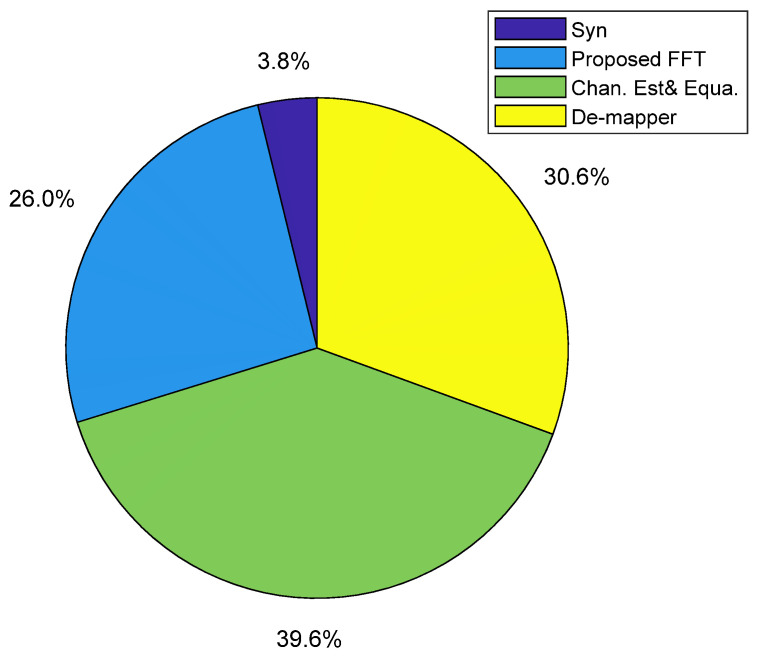
Updated power distribution.

To evaluate the energy efficiency of the proposed FFT, the 16-bit quantized Spiral FFT serves as the power consumption baseline. The power consumption of both the Spiral FFT and the proposed FFT is measured under various modulation formats (64-QAM, 32-QAM, 16-QAM, and adaptive) across a wide range of received optical powers (−5, −9, −13, −17, and −21 dBm) typical in PON systems. The results, summarized in Figure 10, lead to two key findings. Firstly, the modulation format has a negligible impact on the power consumption of either FFT implementation. Secondly, the energy savings of the proposed FFT increase as the received optical power decreases, reaching 66.5% at −5 dBm and 76.1% at −21 dBm. This trend is a direct consequence of the dynamic bit-resolution scaling in the proposed FFT, which reduces computational precision at lower ROP levels. The average energy saving across all tested conditions is approximately 70%. Leveraging the DSP module power breakdown for the ML605-based receiver [8], Figure 11 illustrates the updated power distribution when the proposed FFT is employed at an ROP of −21 dBm. A key outcome is evident: the FFT module is no longer the dominant consumer power within the FPGA-based real-time IMDD OFDM-PON receiver.

The FPGA resource utilization for the proposed FFT is detailed in Table 4, and the FPGA resource utilization for Spiral FFT with variable quantization bits is shown in Table 5. The results demonstrate that the proposed architecture successfully eliminates the need for high-power block RAM (BRAM), lookup table RAM (LUTRAM), and digital signal processing (DSP) slices, while also substantially reducing the consumption of flip-flop (FF) and lookup table (LUT) resources. Furthermore, the power consumption model (Equation (2)) was applied to the resource utilization data from Table 4 to generate the estimated power curve in Figure 10. The close agreement between this estimation and the empirically measured power consumption provides strong additional validation for both the accuracy of the power model and the efficacy of the proposed low-power design.

### 5.3. Real-Time Experimental Verification of the Energy-Efficient Effect of the Overall Energy-Efficient Scheme

**Figure 12:**
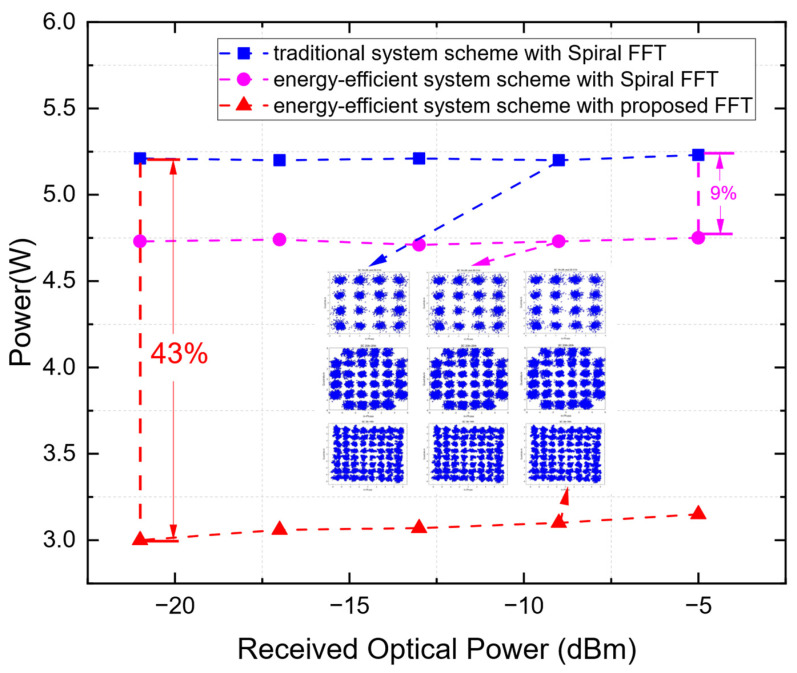
Power Consumption of Different Schemes.

The overall energy savings achieved by the system-level solution for the FPGA-based real-time IMDD OFDM-PON receiver are presented in Figure 12, with all measurements conducted using adaptive modulation. The system-level solution alone, when applied with the Spiral FFT, yields an energy saving of approximately 9% across the tested ROP range. When combined with the proposed FFT, the total energy saving reaches 43% at an ROP of −21 dBm. This enhanced saving arises from the synergistic effect of the two strategies: the system-level solution reduces the clock rate for downstream modules, while the proposed FFT itself consumes less power, a reduction that is further amplified at lower ROP levels due to its dynamic bit-resolution scaling.

## 6. Conclusions

In this paper, a highly energy-efficient 32-parallel 64-point FFT scheme for FPGA-based real-time IMDD OFDM-PON is experimentally demonstrated for the first time on a Xilinx ML605 platform, achieving up to 70% power reduction for high-parallel FFT. A key enabler is the development and experimental validation of a power consumption model that establishes a clear relationship between FFT power dissipation and FPGA logic resource utilization. This model leads to the derivation of a resource selection principle aimed at minimizing power consumption. By applying this principle through dynamic bit-resolution scaling tailored to the received optical power, the proposed FFT achieves a 76.1% reduction in power consumption compared to the traditional Spiral FFT at −21 dBm. Consequently, the FFT module is no longer the dominant power consumer within the DSP chain of the real-time IMDD OFDM-PON system, marking a significant shift in the system’s power profile.

## Figures and Tables

**Table 1:** Power consumption coefficients for Xilinx Virtex-6 FPGA.

Parameters	Value
α	2.08 × 10^−5^ W/MHz
β	2.63 × 10^−5^ W/MHz
η	7.69 × 10^−5^ W/MHz
θ	4.42 × 10^−3^ W/MHz
δ	9.73 × 10^−4^ W/MHz

**Table 2:** Key system parameters.

Parameters	Value
FFT/IFFT points	64
Data-carrying subcarriers	From 2 to 28
Modulation format	2/4/8/16/32/64-QAM
ADC/DAC resolution	10/12-bit
ADC& DAC sample rate	4 GS/s
OFDM frame CP	16 samples (4 ns)
PRBS	2^15^ − 1
Transmitter output power	+7.75 dBm
DFB wavelength	1549.98 nm
DFB modulation bandwidth	2.7 GHz
DFB bias current	45 mA
DFB driving voltage	2 Vpp
PIN detector bandwidth	40 MHz~3 GHz
PIN responsivity	0.9 mA/mW

**Table 3:** The Minimum Required Bit Width.

Receive Optical Power	Stage Index					
	1	2	3	4	5	6
−5 dBm	11	12	11	11	11	11
−9 dBm	11	12	10	11	10	11
−13 dBm	11	12	10	10	10	10
−17 dBm	11	12	9	10	9	10
−21 dBm	11	12	8	9	8	9

**Table 4:** OFDM-PON System Parameters.

Received Optical Power	FF/LUT/LUTRAM/BRAM/DSP
−5 dBm	10,816/9855/0/0/0
−9 dBm	10,424/9639/0/0/0
−13 dBm	9896/9231/0/0/0
−17 dBm	9482/8949/0/0/0
−21 dBm	8602/7789/0/0/0

**Table 5:** The resource usage of the Spiral FFT for variable quantization bits.

Quantization Bits	FF/LUT/LUTRAM/BRAM/DSP
16	19,813/12,855/2840/96/172
15	14,368/11,876/2020/48/172
14	13,385/11,203/2007/48/172
13	12,402/10,567/1997/48/172

## Data Availability

The raw data supporting the conclusions of this article will be made available by the authors on request.
